# RKIP phosphorylation and STAT3 activation is inhibited by oxaliplatin and camptothecin and are associated with poor prognosis in stage II colon cancer patients

**DOI:** 10.1186/1471-2407-13-463

**Published:** 2013-10-08

**Authors:** Sam Cross-Knorr, Shaolei Lu, Kimberly Perez, Sara Guevara, Kate Brilliant, Claudio Pisano, Peter J Quesenberry, Murray B Resnick, Devasis Chatterjee

**Affiliations:** 1 Department of Medicine, Rhode Island Hospital and The Alpert Medical School of Brown University, Providence, RI 02903, USA; 2 Department of Pathology, Rhode Island Hospital and The Alpert Medical School of Brown University, Providence, RI 02903, USA; 3 Department of Pathology, Sigma Tau Inc, Rome, Italy

**Keywords:** RKIP/pRKIP, STAT3, IL-6, CPT, OXP, TMA, Stage II colon cancer, LVI, Luciferase reporter assay

## Abstract

**Background:**

A major obstacle in treating colorectal cancer (CRC) is the acquired resistance to chemotherapeutic agents. An important protein in the regulation of cancer cell death and clinical outcome is Raf kinase inhibitor protein (RKIP). In contrast, activated signal transducer and activator of transcription 3 (STAT3) is a protein that promotes tumor cell survival by inhibiting apoptosis and has an important role in cancer progression in many of cancer types. The aim of this study was to evaluate the regulation of RKIP and STAT3 after treatment with clinically relevant chemotherapeutic agents (camptothecin (CPT) and oxaliplatin (OXP)) and the cytokine interleukin-6 (IL-6) in HCT116 colon cancer cells as well as evaluate the association between RKIP and STAT3 with clinical outcome of Stage II colon cancer patients.

**Methods:**

HCT-116 colon cancer cells were treated with CPT, OXP, and IL-6 separately or in combination in a time and dose-dependent manner and examined for phosphorylated and non-phosphorylated RKIP and STAT3 via Western blot analysis. STAT3 transcriptional activity was measured via a luciferase reporter assay in HCT116 cells treated with CPT, IL-6 or transfected with JAK 1, 2 separately or in combination. We extended these observations and determined STAT3 and RKIP/ pRKIP in tumor microarrays (TMA) in stage II colon cancer patients.

**Results:**

We demonstrate IL-6-mediated activation of STAT3 occurs in conjunction with the phosphorylation of RKIP *in vitro* in human colon cancer cells. OXP and CPT block IL-6 mediated STAT3 activation and RKIP phosphorylation via the inhibition of the interaction of STAT3 with gp130. We determined that STAT3 and nuclear pRKIP are significantly associated with poor patient prognosis in stage II colon cancer patients.

**Conclusions:**

In the analysis of tumor samples from stage II colon cancer patients and the human colon carcinoma cell line HCT116, pRKIP and STAT3, 2 proteins potentially involved in the resistance to conventional treatments were detected. The phosphorylation of pRKIP and STAT3 are induced by the cytokine IL-6 and suppressed by the chemotherapeutic drugs CPT and OXP. Therefore, these results suggest that STAT3 and pRKIP may serve as prognostic biomarkers in stage II colon cancer patients and may improve chemotherapy.

## Background

Globally, (CRC) is the third most common diagnosed cancer in men and second in women {Jemal, 2011 #316}. With the annual worldwide incidence rate of colon cancer rising to over 1.2 million in 2008, up from less than 0.95 million in 2005, the number of annual deaths has also risen by 100,000 in the same three-year span [1,2]. Surgical resection is the only curative treatment option for local regional disease. Clinical outcome is dependent upon extent of disease at presentation, also known as tumor stage. Five-year survival rates according to tumor stage at diagnosis based on the patient data collected in the SEER database between 1991 and 2000 were as follows: 72-85% for stage II patients, 44-83% for stage III patients, and 8% for stage IV [3]. For patients that have undergone potentially curative resection (stage II and III patients), disease recurrence has been attributed to clinically occult micro-metastases present at the time of surgery, which are targeted by postoperative therapy. However, despite multi-modality therapy, survival rates are still modest. As a result multiple hypotheses have been developed to account for the limitations in current treatment modalities. One argument described discusses the impact of genetic aberrations that arise during the development of CRC, which can lead to a reduced susceptibility to apoptosis which could account for the resistance to chemotherapy [3-5].

Raf kinase inhibitor protein (RKIP) is a member of the phosphatidylethanolamine-binding protein family [6] and is an inhibitor of the mitogen-activated protein kinase cascade initiated by Raf-1 [7]. RKIP can affect various diseases including cancer, Alzheimer’s disease, and pancreatitis, which makes it a logical target for individualized therapy and disease-specific interventions [8]. The antagonizing effects of RKIP on cell survival also extends to the NF-κB (Nuclear Factor kappa B) [9] and GRK2 (G Protein-Coupled Receptor Kinase 2) pathways [10]. RKIP is induced upon exposure to many chemotherapeutic agents and plays a key role in the apoptosis of tumor cells [11]. Studies have shown that when RKIP is phosphorylated on the Ser153 residue by PKC (Protein Kinase C) it is inactivated and subsequently dissociates from Raf-1, therefore ending the inhibition of the Raf-MEK-ERK proliferation pathway [12,13].

STAT family proteins are localized primarily in the cytoplasm, but upon activation (phosphorylation and acetylation) they dimerize and localize to the nucleus to regulate genes involved with cellular growth, proliferation and metastasis [14-16]. STAT3 is phosphorylated on a tyrosine residue (pY705) by Janus kinases (JAKs) [17,18]. Abnormal JAK activity is primarily responsible for the constitutive activation of STAT3 and the development of a tumorigenic phenotype in various cancers, including colon [19-23]. Therefore, disrupting the activation of STAT3 has the potential to enhance chemotherapy induced apoptosis and treatment outcomes.

Interleukin-6 (IL-6) is an inflammatory chemokine released by a variety of cells, including T-cells and macrophages, which binds and signals through the IL-6 receptor and the β-receptor subunit glycoprotein 130 (gp130) [24-26]. IL-6 stimulation through gp130 activates the JAK/STAT pathway, leading to cell proliferation and survival [27,28]. IL-6 has been linked to metastasis into bone [29,30] and elevated IL-6 levels have been observed in various tumors and cell lines [31,32]. Thus, aberrantly high IL-6 levels cause the phosphorylation of STAT3 [19], leading to cancer cell survival [14,22]. In colon cancer, the membrane bound IL-6 receptor expression was found to be decreased, whereas the production of soluble IL-6 receptor was increased, leading to greater STAT activation and the induction of pro-survival proteins [33,34]. IL-6 signaling has been shown to be TGF-beta dependent, where suppression of TGF-beta led to decreased STAT activation and the prevention of *in vivo* tumor progression [33].

Currently, patients with node positive or metastatic colon cancer demonstrate an overall survival benefit when treated with a fluoropyrimidine-based regimen. Colon cancer patients with metastatic disease receiving an OXP combination chemotherapy are about twice as likely to respond to treatment compared to the same drug combinations without OXP [35,36]. It has also been demonstrated that these patients survive longer [36]. Over the last decade, similar fluoropyrimidine combinations have been evaluated in patients with node positive disease, and unlike patients with metastatic colon cancer, improvement in clinical outcome was only demonstrated in regimens of a fluoropyrimidine alone or in combination with OXP, also referred to as FOLFOX. [37,38]. Unfortunately, the survival benefits of patients treated with a combination of 5-fluorouacil leucovorin, and, the CPT analog, irinotecan (a combination known as FOLFIRI) is restricted to stage IV colon cancer, [3] and the response rate in this patient population is roughly about 50% [36,39]. The benefits of FOLFOX post-operative systemic therapy has been clearly demonstrated in stage III disease, the value in stage II is small but present; and on subgroup analysis, patients with high-risk stage II tumors demonstrated a trend toward improved disease free survival. Current standard, supported by the National Comprehensive Cancer Network (NCCN) is FOLFOX and consists of 5-fluorouracil, leucovorin, and oxaliplatin (OXP) [38,40].

OXP is a derivative of cisplatin that is able to cause apoptosis in cells previously resistant to cisplatin [41]. Apoptotic signaling is initiated when OXP binds to DNA, forming a DNA adduct [40]. Camptothecins (CPTs) are another class of chemotherapeutic compounds used clinically to treat various malignancies including metastatic CRC. Camptothecin and its congeners target the enzyme topoisomerase 1 by binding to the DNA-Top1 complex and preventing the replication of DNA [42]. Camptothecin derivatives can induce RKIP expression and apoptosis in some human cancer cells [11].

One major obstacle in elongating the post-treatment survival of patients after conventional therapies, such as radiation and chemotherapeutic drugs like OXP and CPT, is the acquired resistance observed in many patients with colon cancer [43-45]. One way to understand the mechanism by which this resistance arises is to analyze how the drug modulates proteins involved with survival and apoptosis. Therefore, it is necessary to find specific gene and protein targets to help improve the outcome of colon cancer treatment. Recent reports indicate that RKIP may serve as a potential biomarker in Dukes’ B CRC patients and used to identify ‘high-risk’ patients with aggressive CRC and these patients should be considered for adjuvant therapy, which may be dependent on intratumoural heterogeneity [46,47].

In this study we demonstrate that IL-6 mediated activation of STAT3 occurs in conjunction with the phosphorylation of RKIP *in vitro*. OXP and CPT are able to block the IL-6 mediated STAT3 activation and RKIP phosphorylation via the inhibition of the interaction of STAT3 with gp130. We extended these observations and determined that that STAT3 and nuclear pRKIP are associated with poor patient prognosis in stage II colon cancer patients.

## Methods

### Materials

The CPT derivative ST2614 was provided by Sigma Tau Inc., Rome, Italy. Recombinant human IL-6 was purchased from BD Pharmingen Biosciences. All other reagents and chemicals were purchased from Sigma Chemical Co. unless otherwise noted. Protein quantification reagents were obtained from Bio-Rad Laboratories Inc. and Thermo Scientific. Enhanced chemiluminescence reagents and secondary mouse and rabbit antibodies conjugated to horseradish peroxidase for Western blot analysis were from GE Healthcare. The antibodies to STAT3 (sc-482), pRKIP (sc-32623), gp130 (SC-655) and actin (SC-1616) were purchased from Santa Cruz Biotechnology; STAT3 pY705 (9131S) and PARP (9542S) from Cell Signaling Technology; RKIP (07–137) and Histone 2AX (07–67) from Millipore, Milford, MA.

### Cells and plasmid

The human colon cancer cell lines, HCT116 and HT29 were purchased from ATCC (Rockville, MD). The HCT116 cells were grown in McCoy’s 5A and HT29 cells in RPMI1640 medium (Invitrogen) supplemented with 10% fetal bovine serum, glutamine, non-essential amino acids, 100 units/ml penicillin, and 100 μg/ml streptomycin. They were cultured in a humidified incubator at 37°C containing 5% CO_2_.

### Western blot analysis

Total cell extracts were prepared as previously reported [11] and the protein concentrations of lysates were determined using either Bradford assay kit (BioRad) or BCA protein assay kit (Pierce). Proteins were separated by 10% SDS-PAGE and electrophoretically transferred from the gel to nitrocellulose membranes (GE Healthcare). Proteins recognized by antibodies were detected by enhanced chemiluminescence (ECL) reagents (GE Healthcare).

### Annexin V apoptosis analysis

HCT116 cells were plated at 3 X 10^5^ and treated with the appropriate agent for the indicated times. Cells were harvested with 0.25% trypsin (Invitrogen) and the PE Annexin V Apoptosis Kit 1 (BD Pharmingen) was used according to the manufacturer’s protocol to measure early and late stage apoptosis. Cells that stained positive for both 7-AAD and PE Annexin V (7+ and PE+) are in late stage apoptosis whereas those that stain PE+, but 7- are still in the early stages of apoptosis. Staurosporine was used as a positive control of apoptosis.

### Transfection of HCT116 cells

Cells were transiently transfected using the Lipofectamine transfection reagent (Invitrogen) according to the manufacturer’s protocol. Total DNA quantities of 1 or 2 μg were transfected per sample.

### STAT3 luciferase reporter assay

Cells (3 x 10^5^ cells/60 mm dish) were transiently transfected with 0.25 μg of a reporter plasmid containing STAT3 binding fragments of the promoter region of mouse IRF1 gene using lipofectamine in serum-free medium [14]. After 3 hours, OPTI-MEM containing FBS (fetal bovine serum) was added to the cells at a final concentration of 20% FBS. Cells were harvested by scraping, washed twice with PBS and lysed in passive lysis buffer (Promega). The luciferase activity in the cytosolic supernatant was evaluated using the Dual Luciferase Reporter Assay (Promega) and measured using a luminometer (Lumat LB 9507, Berthold Technologies) to estimate transcriptional activity.

### Immunoprecipitation assay

Cells were transfected with an empty vector (EV) or indicated plasmids for 48 h. In experiments exploring CPT, cells were treated at 200 nM for 16 h. Samples were lysed in RIPA buffer with complete protease inhibitors (Roche). Approximately 5% of the sample was removed for total protein analysis of the immunoprecipitaion (IP) input. The remainder of the sample, 1.5 mg of protein, was incubated with monoclonal HA antibody and placed on a rotator for 4 h at 4°C. Immunocomplexes were isolated with protein G-agarose beads, separated by 10% SDS-PAGE, and electroblotted to a nitrocellulose membrane. Proteins were detected via incubation with the indicated antibodies and an ECL detection system.

### Patients and specimens

Archival cases of Stage II colorectal adenocarcinoma from 140 consecutive patients were collected between the years of 1986 to 2005 from the archives of the Department of Pathology at the Rhode Island Hospital. Stage was defined according to American Joint Committee on Cancer criteria [48]. None of these patients received adjuvant chemotherapy or radiotherapy before surgery or after the initial resection. Recurrence and survival data were ascertained through the Rhode Island Tumor Registry and Rhode Island Hospital chart review. The Institutional Review Board at the Rhode Island Hospital approved this study. All tissue samples were formalin fixed and paraffin embedded. The corresponding H&E slides were reviewed for confirmation of diagnosis and adequacy of material by SL and MR.

### Tissue microarray (TMA) construction

Paraffin blocks containing areas consisting of invasive colon carcinoma were identified on corresponding H&E-stained sections as previously described [49]. Areas of interest that represented non-necrotic invasive front of the adenocarcinoma were identified and marked on the source block. The source block was cored, and a 1-mm core was transferred to the recipient “master block” using the Beecher Tissue Microarrayer (Beecher Instruments). Three to six cores of tumor were arrayed per specimen. In addition, a core of normal adjacent colonic mucosa was also sampled when present.

### Immunohistochemistry

Immunohistochemistry for each antigen was done on 5-μm-thick paraffin sections of colon cancer tissue microarray sample described above. The microarrays were immunohistochemically stained for phosphorylated RKIP and a full-length STAT3 antibody (polyclonal rabbit; 1:150; Santa Cruz Biotechnology, Inc.) using the Ventana Discovery automated system using the DABMAP and CC1 antigen retrieval (Ventana Medical Systems, Inc.). Slides were dehydrated, cleared, and mounted. Positive controls consisted of multitumor and normal tissue microarrays generated in our department. Negative controls included replacement of the primary antibody with non-reacting antibodies of the same species.

### Quantitative immunohistochemical analysis

The nuclear and the cytoplasmic staining patterns were separately quantified, for both phosphorylated RKIP and STAT3, using a semiquantitative system for evaluation and grading of the immunostaining pattern, successfully applied by us and others [50,51]. The phosphorylated RKIP (nuclear and cytoplasmic) staining intensity was scored into four categories: 0 for complete absence of the staining, 1 for weak staining, 2 for moderate, and 3 for strong staining. The extent of the positively stained cells was also scored into a percentage. Each core was given a score derived from the calculation of grade −1 + percentage/100 (e.g. 1.5 is the final score of a grade 2 with 50% positive area). Score of each case is the average of all the cores of the case. At least three cores were scored per case. The STAT3 staining intensity was scored in the same fashion. The score ranges from 0 to 3. This scoring system takes both intensity and extension into consideration. To convert it into a more understandable quantile format, scores of 0 are graded as 0, scores >0 and < =1 are graded as 1+, scores >1 and < =2 are graded as 2+, and scores >2 are graded as 3+. All sections were scored independently by SL and were blinded to the clinicopathologic features or clinical outcome.

### Statistical analysis

Chi-square analysis was used to evaluate the association between STAT3 expression and tumor grade and lymphovascular invasion (LVI) in tumor. All tests were two-sided and p-values of 0.05 or less were considered statistically significant. Statistical analyses were done using the JMP 8.0 statistical program (SAS Institute, Cary, NC). The vast majority of the cases have a complete set of staining data and clinicopathologic information upon which statistical analysis was performed. All cell culture experiments were repeated at least 3 times, unless indicated otherwise, and paired t-tests were used to determine statistical significance.

## Results

### Treatment with IL-6 enhances phosphorylated RKIP levels

**Figure 1:**
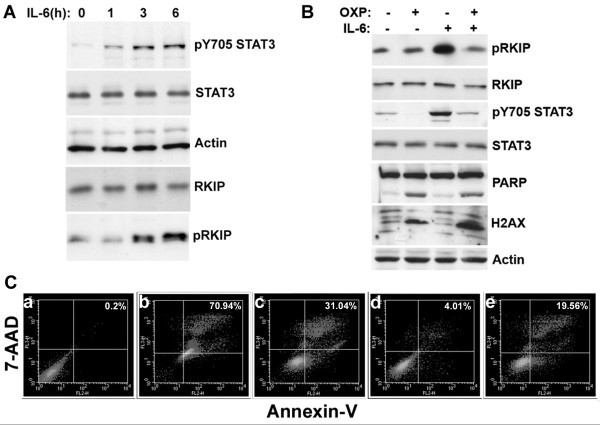
**Oxaliplatin (OXP) reduces the phosphorylation of STAT3 and RKIP that is induced by interleukin-6 (IL-6).** Western blot analysis of: **(A)** the indicated proteins in HCT116 cells treated with IL-6 (40 ng/ml) for 1–6 h. **(B)** HCT116 cells treated with 300 μM OXP, IL-6 (40 ng/ml) in serum free medium or the combination for 16 h and analyzed for the indicated proteins via Western blot analysis. **(C)** Cell extracts were prepared after 18 h following treatment with OXP, IL-6 or the combination for flow cytometric analysis to examine binding to 7-AAD and annexin-V (% Apoptosis). The % Apoptosis of each sample is indicated in the top right corner of every panel: **a)** untreated control cells (0.20%); **b)** 5 μM STS (70.94%); 300 μM OXP (31.04%); **d)** IL-6 (4.01%); **e)** OXP + IL-6 (19.56%). STS is the positive control. The figure is representative of part of 1 experiment performed in duplicate. The experiment was repeated twice.

IL-6 has been shown to lead to STAT3 activation in colon cancer [27,28]. HCT116 cells were treated for 1, 3 and 6 h with 40 ng/ml IL-6 and examined for STAT3 and RKIP phosphorylation. As expected, we observed an increase in pY^705^STAT3 but were surprised to also note an increase in pRKIP (Figure 1A). To our knowledge this is the first report to show cytokine-mediated phosphorylation of RKIP.

### Oxaliplatin inhibits IL-6 signaling

Previous studies have shown that treating CRC CT26 cells with 300 μM OXP for 24 h leads to about 50% of the cells showing signs of apoptosis [52]. In our experiment treatment with OXP induced approximately 32% of the cells to undergo apoptosis, which was lowered to 19% after co-treatment with IL-6 (Figure 1C). Western blot analysis showed that co-treatment of HCT116 cells with IL-6 and 300 μM OXP for 18 hours inhibited the increase in pY^705^ STAT3 and pRKIP caused by IL-6 (Figure 1C). OXP induced apoptosis was confirmed with Western blot analysis by measuring PARP (Poly-ADP-ribose polymerase) cleavage and DNA damage by H2AX (Histone 2AX) phosphorylation [11,53,54] (Figure 1B).

### CPT (ST2461) reduces IL-6 induced RKIP phosphorylation and STAT3 transcription

**Figure 2:**
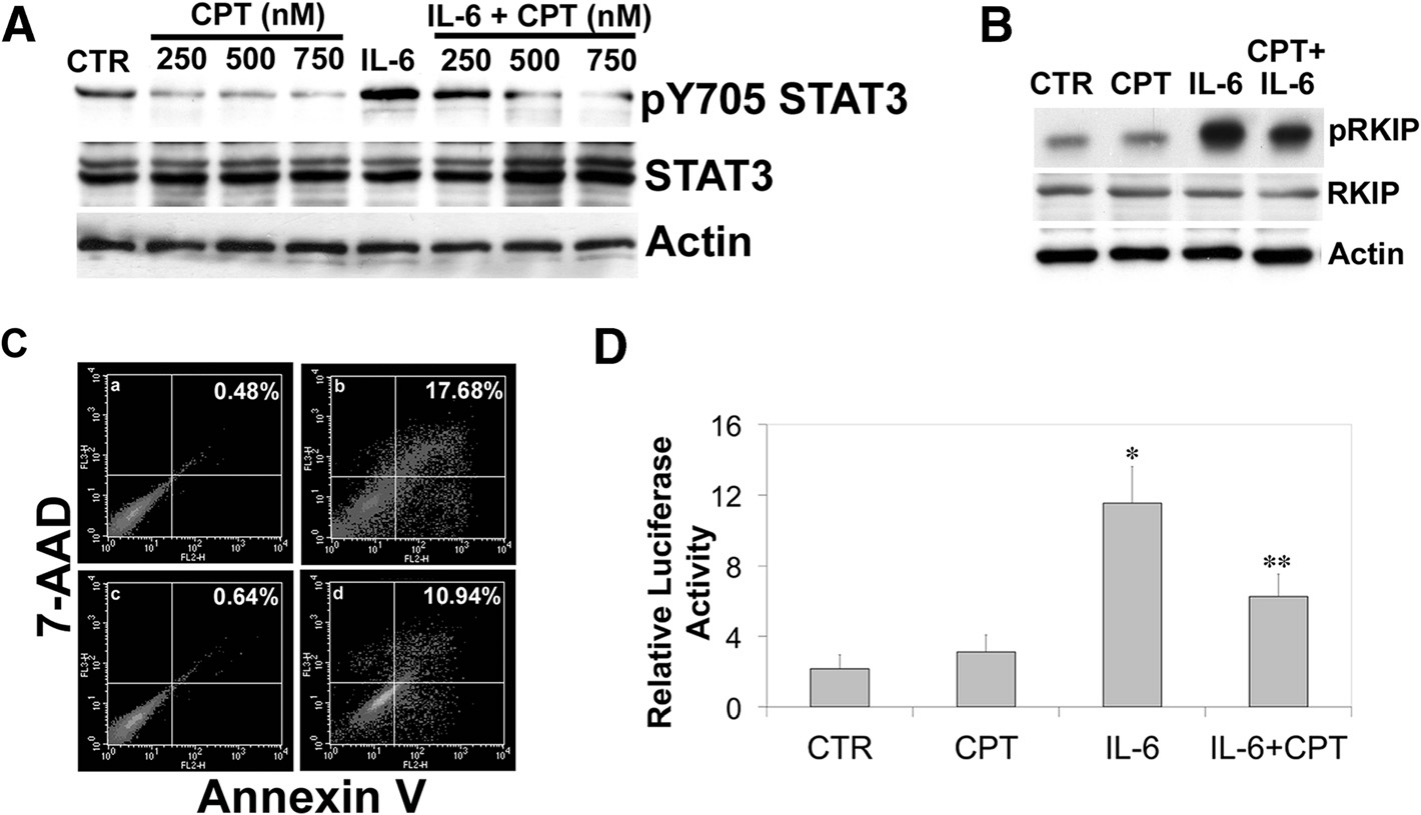
**Camptothecin (CPT) reduces phosphorylation of STAT3 and RKIP induced by interleukin-6 (IL-6). (A)** Western blot analysis of: a dose-dependent reduction of STAT3 phosphorylation after IL-6 treatment then treated with 250–750 nM CPT; HCT116 cells treated with 250 nM CPT and 40 ng/ml IL-6 for 12 h. **(B)** The same co-treatment experiment as in **(A)** was repeated with HCT116 cells treated with 250 nM CPT and 40 ng/ml IL-6 for 12 h. Western blot analysis was performed to examine the protein levels of pRKIP, RKIP and actin. **(C)** Cell extracts were prepared after 18 h following treatment with CPT, IL-6 or the combination for flow cytometric analysis to examine binding to 7-AAD and annexin-V (% Apoptosis). The % Apoptosis of each sample is indicated in the top right corner of every panel: **a)** untreated control cells (0.48%); **b)** CPT (17.68%); **c)** IL-6 (0.64%); **d)** CPT + IL-6 (10.94%). The figure is representative of part of 1 experiment performed in duplicate. The experiment was repeated twice. **(D)** HCT116 cells were transfected with an IRF-1 reporter plasmid for STAT3 activation. After 48 h, the cells were washed and treated with 40 ng/ml IL-6, 250 nM CPT, or the combination. After 24 h, samples were harvested and washed twice before being lysed and combined with a luciferase assay reporter. The data is reported as the mean +/− s.d. of 2 independent experiments performed in triplicate. A paired t-test was performed to analyze the increase in STAT3 transcription of IL- 6 treated experimental samples when compared to vehicle (CTR): *IL-6, p < 0.000012; or decrease when comparing IL-6 to samples treated with **IL-6 and CPT, p < 0.0002.

Camptothecin is frontline therapy for metastatic CRC [3]. Therefore, we investigated if CPT could affect STAT3 phosphorylation. Western blot analysis revealed a dose-dependent decrease of STAT3 pY705 phosphorylation when cells were treated with 40 ng/ml IL-6 in the presence of 250–750 nM CPT for 12 h (Figure 2A). The same experiment was repeated and the cells were treated with 250 nM CPT and 40 ng/ml IL-6. We observed a reduction of pRKIP when the cells were treated with both compounds (Figure 2B). We measured apoptosis in the samples via Annexin staining from Figure 2 B and found that treatment with 250 nM CPT led to approximately 17% of the cells to undergo apoptosis, which was reduced to 7% after co-treatment with IL-6 (Figure 2C). STAT3 luciferase reporter assay confirmed a significant decrease (p < 0.0002) in STAT3 transcription when cells were treated with IL-6 and CPT (Figure 2D). We found that these effects were also recapitulated in HT29 colon cancer cells (Additional file 1: Figure S1). In addition to inhibiting TOP I, this CPT analogs can also interfere with cytokine-mediating signaling events that lead to RKIP and STAT3 phosphorylation.

### STAT3 overexpression increases pRKIP

**Figure 3:**
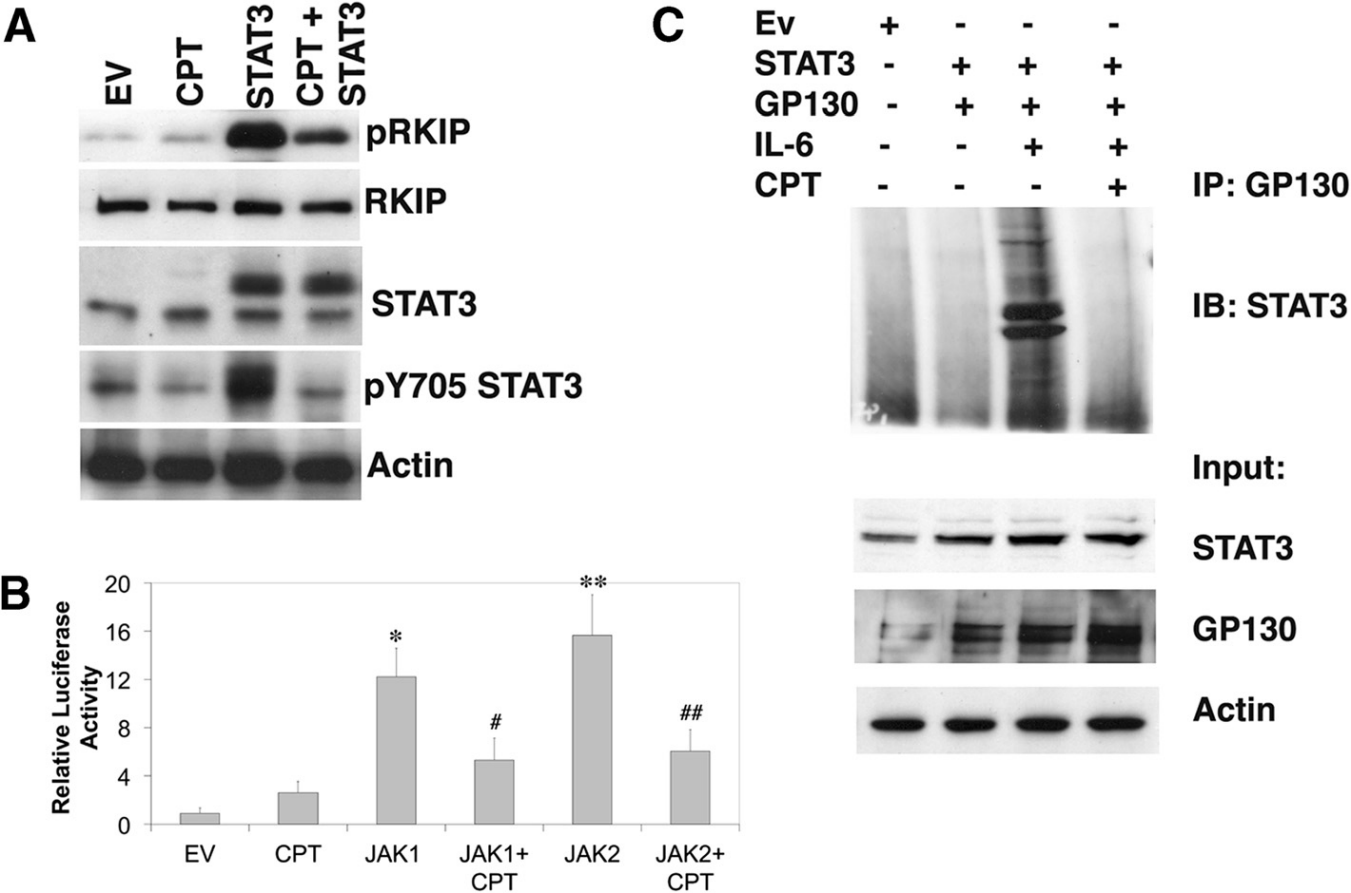
**Camptothecin blocks STAT3 activation and the interaction of STAT3 and the gp130 receptor. (A)** Western blot analysis for the indicated proteins from HCT116 cells transfected with STAT3 cDNA and then treated with 250 nM CPT for 16 h. **(B)** HCT116 cells were transfected with an IRF-1 reporter plasmid to measure STAT3 activation along with JAK1 and 2 cDNAs. After 48 h, cells were washed and treated 250 nM CPT. After 24 h, the samples were harvested and washed twice before being lysed and combined with a luciferase assay reporter. The data is reported as the mean +/− s.d. of 2 independent experiments performed in triplicate. **(C)** HCT cells were transfected with STAT3 cDNA and gp130 cDNA. After 48 h, Samples were treated with 40 ng/ml IL-6 or IL-6 and 250 nM CPT. Samples were divided and either saved for Western blot analysis (input) or incubated with an antibody to gp130 for 6 h. Protein G agarose beads were added and the samples rotated over night. Western blot analysis was performed using the IP supernatant and examined for the indicated proteins. In comparison to empty vector controls (EV), the relative activity of STAT3 transcription was increased by: *JAK1, p < 0.00005; **JAK2, p < 0.0001. In the presence of CPT, JAK1-mediated STAT3 transcription was inhibited #JAK1 + CPT p < 0.0002 and JAK2 inhibited ## JAK2 + CPT p < 0.0003. The data represents the mean +/− s.d. of 2 independent experiments performed in duplicate.

IL-6 treatment enhances STAT3 phosphorylation, transcription and pRKIP (Figures 1 and 2). We examined if STAT3 overexpression could directly affect pRKIP and Western blot analysis showed that the expression levels of phosphorylated RKIP increased upon transfection with STAT3 (Figure 3A). In the presence of CPT, the levels of pRKIP were reduced after STAT3 overexpression (Figure 3A) when compared to STAT3 alone (Figure 3A). This indicates, similar to our IL-6 results (Figure 2) that CPT interferes with the kinase activity mediated by STAT3 that results in RKIP phosphorylation.

### JAK induced transcription of STAT3 is inhibited by CPT

In order to further examine the disruptive effects of CPT on HCT116 cells proliferation signaling we performed various luciferase assays to measure STAT3 transcription. JAK proteins are known to enhance STAT3 transcription [17,18], thus we measured the effect of CPT on JAK-mediated STAT3 transcription. We found that STAT3 transcriptional activity is significantly increased in cells transfected with JAK1 (p < 0.0005) and JAK2 (p < 0.0001) (Figure 3B). However, the addition of CPT decreased JAK1 (p < 0.0002) and JAK2 (p < 0.0003)-mediated STAT3 transcription (Figure 3B).

### CPT diminishes pRKIP levels through the inhibition of STAT3 by interacting with GP130

To delineate the observed changes in pY^705^ STAT3 levels after CPT treatment we performed an immunoprecipitation assay. Western blot analysis revealed that the interaction between gp130 and STAT3 is IL-6 dependent and that this interaction is interrupted by CPT treatment (Figure 3C). This indicates that treatment with CPT leads to the disruption of subsequent phosphorylation events after IL-6 treatment. Collectively our results (Figures 1, 2 and 3) suggest that CPT affects multiple pathways leading to diminution of kinase activities.

### Clinicopathologic features of cancer patients luciferase reporter assay luciferase reporter assay

To see if we could correlate our cell-based studies with the colon cancer patient clinical outcome we examined a TMA of 140 patients. The mean age of the patients at initial surgery was 74.3 years (range, 30–97 years); 66 men and 74 women were included in the study. The mean duration of follow-up was 76.6 months (range, 16–250 months). All the tumors were Stage II with 25 cases of high grade and 115 cases of low grade based on the latest American Joint Committee of Cancer tumor stage [48]. There were 13 tumors with LVI (lymphovascular invasion) and 127 tumors without LVI. The clinicopathologic features of the patients are summarized in Table 1.

**Table 1:** The clinicopathologic features of the patients studied

**Patient characteristics (n = 140)**	
Feature	Frequency (n,%)
Gender	
Male	66 (47.1)
Female	74 (52.9)
Age (years)	
Mean (min, max)	74.3 (30–97)
Tumor size (mm)	
Mean (min, max)	5.17 (1, 11)
Tumor grade	
Low	25 (17.9)
High	115 (82.1%)
Lymphovascular invasion	
Present	13 (9.3)
Not present	127 (90.7)
Recurrence	
Yes	19 (13.7)
No	120 (86.3%)
Average follow-up time (mean, range)	
76.6 months (16–250)	

All patients were diagnosed with Stage II colorectal adenocarcinoma. Samples were collected between the years of 1986 to 2005 from the archives of the Department of Pathology at the Rhode Island Hospital.

### Expression of phosphorylated RKIP in colon cancer and its prognostic value

**Figure 4:**
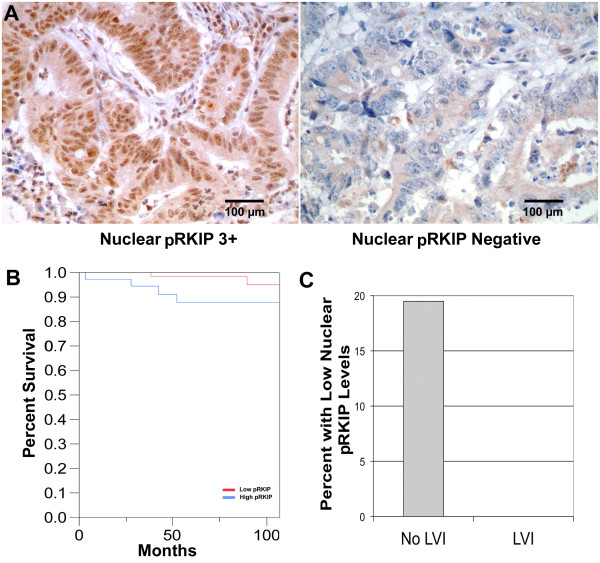
**Phosphorylated RKIP in stage II colon cancer is associated with poor prognosis. (A)** Representative examples of immunohistochemical staining for pRKIP showing strongly positive (3+) and negative levels. **(B)** Kaplan-Meier plot comparing the recurrence-free survival of patients with high versus low levels of nuclear pRKIP. Patients with lower levels experienced significantly longer recurrence-free survival (p = 0.0268). Bar is 100 micron. **(C)** Graphic representation of the correlation between lymphovascular invasion and pRKIP levels (p = 0.03).

The staining pattern for pRKIP is mixed, both cytoplasmic and nuclear (Figure 4A). The cytoplasmic staining intensity was graded 3+ in 66 cases (51.5%), 2+ in 46 cases (35.9%), 1+ in 14 cases (10.9%) and 0 in 2 cases. The nuclear staining intensity (Figure 4A) was graded 3+ in one case, 2+ in 26 cases (20.1%), 1+ in 84 cases (65.1%), and 0 in 18 cases (14.0%). Kaplan Meier survival analysis of a limited number of patients indicated a decrease in survival of patients with elevated pRKIP (Figure 4B). The percent of patients with low levels of pRKIP and no LVI was much greater than the population with LVI (Figure 4C).

Cytoplasmic and nuclear pRKIP have opposite association with two important prognostic markers, tumor grade and lymphovascular invasion (LVI). Twenty six percentage (26%) cytoplasmic pRKIP-low (< 3+) tumors are high grade compared with 11% cytoplasmic pRKIP-high (3+) tumors being high grade (P = 0.024) (Table 2). Similarly 11% cytoplasmic pRKIP-low tumors have LVI while 6% cytoplasmic pRKIP-high tumors have LVI (P = 0.29) (Table 2). Thus, low expression of cytoplasmic pRKIP is associated with high tumor grade and presence of LVI, i.e. worse prognosis. In contrast, 19% of nuclear pRKIP-high (1-3+) tumors are high grade as opposed to 11% of nuclear pRKIP-low (0) tumors being high grade (P = 0.399) (Table 2). Similarly, 10% of nuclear pRKIP-high (1-3+) tumors have LVI while 0% of nuclear pRKIP-low tumors have LVI (P = 0.06) (Table 2). In combination, the data suggests a shift of pRKIP from cytoplasm to nuclei in the process of tumor progression.

**Table 2:** Contingency analysis of cytoplasmic phosphorylated RKIP (c-pRKIP), nuclear phosphorylated RKIP (n-pRKIP) and nuclear STAT3 (n-STAT3) expression and tumor Grade and LVI status

**Marker**	**Grade**		**p-value**	**LVI**		**p-value**
	Low	High		Absent	Present	
c-pRKIP	n (%)	n (%)	0.024	n (%)	n (%)	0.29
0, 1+, 2+	46 (74%)	16 (26%)		55 (89%)	7 (11%)	
3+	59 (89%)	7 (11%)		62 (94%)	4 (6%)	
n-pRKIP	n (%)	n (%)	0.399	n (%)	n (%)	0.06
0	16 (89%)	2 (11%)		18 (100%)	0 (0%)	
1+, 2+, 3+	90 (81%)	21 (19%)		100 (90%)	11 (10%)	
n-STAT3	n (%)	n (%)	0.064	n (%)	n (%)	0.038
0	19 (95%)	1 (5%)		20 (100%)	0 (0%)	
1+, 2+, 3+	86 (80%)	22 (20%)		96 (89%)	12 (11%)	

The numbers and the percentages of cases are provided.

We examined the expression of RKIP in the same cohort of patients and both cytoplasmic and nuclear RKIP staining were evaluated by immunochemistry. However, no statistically significant associations were detected between RKIP expression level (high (2+ and 3+) versus low (0 and 1+)) and tumor grade (p = 0.9191 for cytoplasmic RKIP and p = 0.1918 for nuclear RKIP). Similarly, no statistically significant associations were found between RKIP expression level and LVI (p = 0.1779 for cytoplasmic RKIP and p = 0.1897 for nuclear RKIP). In this study, increased levels of RKIP was inversely associated with tumor grade and high levels of nuclear RKIP was associated with worse prognosis. These results suggest the inactivation of RKIP function possibly via degradation [12], mutation or other mechanisms in Stage II CRC.

### Expression of STAT3 in colon cancer and its association with tumor grade and LVI

**Figure 5:**
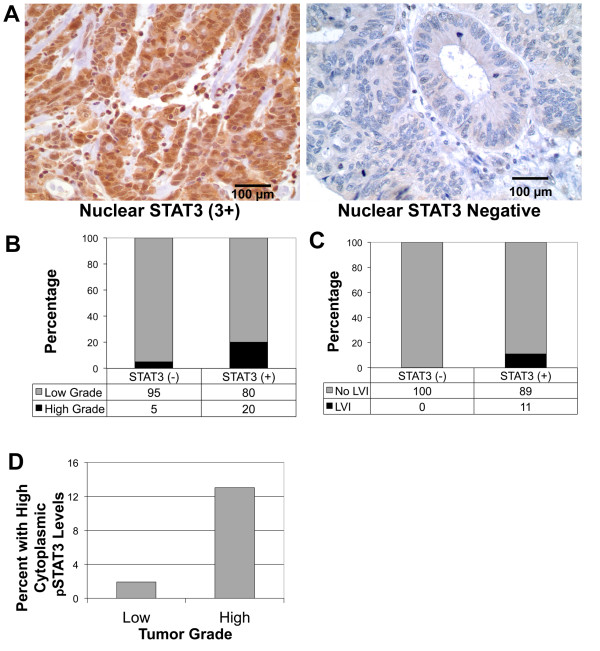
**Nuclear STAT3 in stage II colon cancer is associated with negative pathological features. (A)** Representative examples of immunohistochemical staining for STAT3 showing strongly positive (3+) and negative levels. Bar is 100 micron. **(B)** Nuclear STAT3 is associated with higher grade tumors, where fewer than 5% of high grade tumors received a negative staining score compared to nearly 22% of low grade tumors (p < 0.0266). **(C)** Nuclear STAT3 is associated with lymphovascular invasion. No patients with LVI had negative nuclear STAT3 staining (p < 0.0218). **(D)** High levels of cytoplasmic STAT3 are associated with tumor grade, with higher levels correlating with higher grade (p = 0.03).

STAT3 expression in colon cancer is mainly nuclear (Figure 5A). The nuclear staining intensity (Figure 5A) was graded 3+ in 7 cases 5.5%), 2+ in 45 cases (35.2%), 1+ in 56 cases (43.8%) and 0 in 20 cases (15.6%). The impact of nuclear-STAT3 levels on tumor grade was studied and a significantly greater percentage of nuclear-STAT3 positive tumors are high grade (20%) compared to nuclear STAT3 negative tumors (5%) (p = 0.064) (Table 2 and Figure 5B). Five percent (5%) of nuclear STAT3-negative tumors are high grade, however, 20% of nuclear STAT3-positive (1-3+) tumors are high grade (P = 0.064) (Figure 5B, Table 2). Therefore, nuclear-STAT3 levels are associated with LVI. None of the nuclear STAT3-negative tumors have any LVI while 10% of nuclear STAT3-positive tumors have LVI (P-0.038) (Figure 5C, Table 2). Our results indicate that nuclear STAT3 expression may be associated with worse prognosis. Additional analysis of an increased cohort of patients will be required to definitively determine this. Our results indicate that an increased level of cytosolic pSTAT3 is associated with higher tumor grade (p = 0.03) (Figure 5D, Table 2).

## Discussion

Recent studies show that RKIP levels are an important predictor of tumor progression by measuring RKIP levels at the tumor-front and in tumor budding [55,56]. Phosphorylated RKIP has been shown to be required to promote gastric cancer progression after infection with *Helicobacter pylori*[13]. However, few studies have investigated the role of phosphorylated RKIP and its ability to predict patient outcome. Huerta-Yepez et al. found a significant correlation between pRKIP levels and non-small cell lung cancer patient survival. This was the first study to focus on the clinical significance of pRKIP, revealing that normal levels of pRKIP are associated with better prognosis than low levels [51]. In contrast, our current study indicates that reduced pRKIP may be associated with enhanced survival of stage II colon cancer patients. There may be several reasons for the discrepancies between the studies including that the studies were performed on different tissue types. The phosphorylation of pRKIP may lead to the activation of distinct pathways (i.e., cell survival, apoptosis, anoikis, etc.) in the 2 models, resulting in either better or worse patient prognosis. Here we show the inhibition of pRKIP by CPT and OXP, 2 frontline chemotherapeutic agents used for the treatment of colon cancer patients (Figures 1 and 2), had the opposite correlation between pRKIP levels and patient outcome in Stage II colon cancer. Stage II colon cancer patients with low levels of nuclear pRKIP experienced longer recurrence-free survival compared to that of patients with high levels (Figure 4).

The interaction between RKIP and Raf-1 has been shown to play an important role in CRC survival by suppressing metastasis through the down-regulation of Raf-1 [57] and the up-regulation of RKIP [58]. Furthermore, when RKIP expression in CRC is down-regulated in the cytoplasm, increased vascular invasion and poor patient prognosis are observed [58]. Significantly, RKIP, peritoneal invasion and LVI provide independent prognostic information in Dukes’ B CRC patients [46]. As previously shown, increased expression of RKIP in breast and prostate cancer cells leads to increased sensitization to chemotherapeutic agent as measured by CPT induced apoptosis [11], a similar mechanism may explain the role of RKIP in the resistance to chemotherapeutic agents in CRC patients. Another mechanism of therapeutic resistance relating RKIP to the KEAP1/NRF2 pathway has been described [59]. Apoptosis was associated with the RKIP/KEAP1 expression levels in colorectal cancer tissues, providing another mechanism by which diminution of RKIP levels may result in resistance to therapy [8,59].

Previous studies show that protein kinase C (PKC) is responsible for the direct phosphorylation of RKIP [12], our study has demonstrated that cell survival signaling caused by IL-6 leads to phosphorylation of RKIP (Figure 1). Since high IL-6 levels are linked to tumor growth and progression in colon cancer [33,60] it is logical that we also observed increased levels of pRKIP in these patients. The association between IL-6, pRKIP, and patient survival illustrates the necessity for delineating the mechanism to inhibit the phosphorylation. Previously, IL-6 has been shown to activate STAT3 in colon cancer through phosphorylation on the tyrosine 705 residue [27,28]. Our results suggest that IL-6 triggered STAT3 phosphorylation and activation is correlated with the increase in pRKIP and thus the stimulation of the Raf/MEK/ERK survival pathway. Whether IL-6 stimulation leads to the activation of PKC or other kinase pathways leading to RKIP phosphorylation directly or if this event is associated with the phosphorylation of STAT3 is currently under investigation.

Based on our IHC observations, we further investigated the phosphorylation levels of STAT3. IHC analysis revealed that lower levels of nuclear STAT3 are associated with less invasive tumors and the nuclear expression of STAT3 is significantly associated with high-grade tumors and the presence of lymphovascular invasion (Figure 5). Recent studies have demonstrated details about the STAT3 nuclear localization mechanism [61] and have blocked this localization in human multiple myeloma cells [23]. Therefore, blocking STAT3 localization via Crm A, for example, may be an effective approach to inhibit aberrant STAT3 activity resulting in the inhibition of the phosphorylation, dimerization, or nuclear membrane transport mechanism associated with STAT3 relocation resulting in significant disruption of the cell survival signals in colon cancer.

Chemotherapeutic regimens utilized clinically for patients with stage III CRC typically include a fluoropyrimidine and OXP, whereas a fluoropyrimidine backbone with OXP or CPT is given to patients with stage IV disease. Our data demonstrated that cell survival signaling triggered by IL-6 in HCT116 cells is mitigated by OXP and CPT. Western blot analysis of HCT116 cells treated with IL-6 and OXP demonstrated a reduction in both pRKIP and pY^705^STAT3 back to basal levels (Figure 1). The same observations were made using IL-6 combined with CPT (Figure 2). Since the HCT116 cells are not representative of a particular stage of colon cancer, the fact that both OXP and CPT caused similar reductions in phosphorylation suggests that they trigger similar cellular mechanisms while causing apoptosis. These results support an alternative anti-tumor activity mechanism of action for these compounds.

Our data uncovered another mechanism by which an irinotecan analog CPT (ST2461) is able to inhibit IL-6-mediated STAT3 phosphorylation. STAT3 cannot bind to the gp130 subunit of the IL-6 receptor until IL-6 binds to the extracellular side of the receptor (Figure 3). Treatment with CPT disrupted the binding if STAT3 to gp130 in the presence of IL-6. This inhibition of binding explains why STAT3 was no longer phosphorylated upon IL-6 stimulation in the presence of CPT.

In order to further investigate the involvement of the JAK/STAT pathway in enhancing colon cancer cell survival and the mechanism of RKIP phosphorylation, we examined whether JAK 1 and 2 overexpression could stimulate STAT3 activation and thereby negate the inhibitory effects of CPT. JAK 1 and 2 caused an increase in STAT3 transcription, which was associated with an increase in pRKIP. Treatment with CPT was able to significantly reduce the levels of STAT3 transcription activity and the levels of pRKIP (Figure 3). Therefore, the versatility of camptothecin as a front line chemotherapy agent is increased because, in addition to inhibiting topoisomerase I, CPT is able to enhance apoptosis of cancer cells by disrupting survival signaling of the JAK/STAT pathway at the receptor level.

## Conclusions

In summary, this study examines for the first time, the expression profile of RKIP, pRKIP and STAT3 in Stage II colon cancer. Our results strongly suggest the role of pRKIP and STAT3 in the provision of clinically prognostic and therapeutic information. Our data indicate that the current treatment for colon cancer, FOLFOX and FOLFIRI, are both effective in reducing pRKIP levels *in vitro*. Therefore, examining a larger cohort of patients, in the future, will provide additional data for the assessment of pRKIP and STAT3 for the risk for recurrence of colon cancer.

### Consent

Written informed consent was obtained from the patient for the publication of this report and any accompanying images.

## Abbreviations

CPT: Camptothecin; CRC: Colorectal cancer; FOLFIRI: 5-fluorouacil leucovorin and irinotecan; FOLFOX: 5-fluorouracil leucovorin, and oxaliplatin; IL-6: Interleukin-6; JAK: Janus kinases; LVI: Lymphovascular invasion; OXP: Oxaliplatin; PEBP: Phosphatidylethanolamine-binding protein; pRKIP: Phosphorylated RKIP; RKIP: Raf Kinase Inhibitor Protein; STAT3: Signal transducer and activator of transcription 3; TMA: Tissue microarray.

## Competing interests

The authors declare that they have no competing interests.

## Authors’ contributions

All authors have read and approved the final manuscript. SCK, contributed to the design of the study, data interpretation, and manuscript preparation. SL, contributed to the TMA scoring, data analysis, and manuscript writing. KP contributed to the design of the study and manuscript preparation. SG, contributed to the Western Blot analyses and data interpretation. KB, contributed to the data analysis and preparation of figures. CP, contributed to the manuscript writing and reagents. PQ, contributed to the manuscript writing. MBR, contributed to the TMA scoring, data analysis, and manuscript writing. DC, contributed to the original concept, design of the study, data interpretation, and manuscript writing and preparation.

## Pre-publication history

The pre-publication history for this paper can be accessed here:

http://www.biomedcentral.com/1471-2407/13/463/prepub

## Supplementary Material

Additional file 1: Figure S1 Camptothecin blocks IL-6 mediated STAT3 activation in HT29 colon cancer cells. (A) Western blot analysis of the induction of RKIP and STAT3 phosphorylation after IL-6 treatment and subsequent reduction after treatment with 500 nM CPT; HT29 cells were treated with 500 nM CPT, 40 ng/ml IL-6 or the combination. (B) HT29 cells were transfected with an IRF-1 reporter plasmid to determine STAT3 activation. After 48 h, the cells were washed and treated with 40 ng/ml IL-6, 500 nM CPT, or the combination. After 24 h, samples were harvested and washed twice before being lysed and combined with a luciferase assay reporter. The data is reported as the mean +/− s.d. of 2 independent experiments performed in triplicate. A paired t-test was performed to analyze the increase in STAT3 transcription of IL- 6 treated experimental samples when compared to vehicle (CTR): *IL-6, p < 0.000017; or decrease when comparing IL-6 to samples treated with **IL-6 and CPT, p < 0.0008. Click here for file
