# Supplementary figures and images for: RKIP phosphorylation and STAT3 activation is inhibited by oxaliplatin and camptothecin and are associated with poor prognosis in stage II colon cancer patients
Source: BMC Cancer. 2013 Oct 8;13:463. doi: 10.1186/1471-2407-13-463 (PMC3856511; doi:10.1186/1471-2407-13-463)

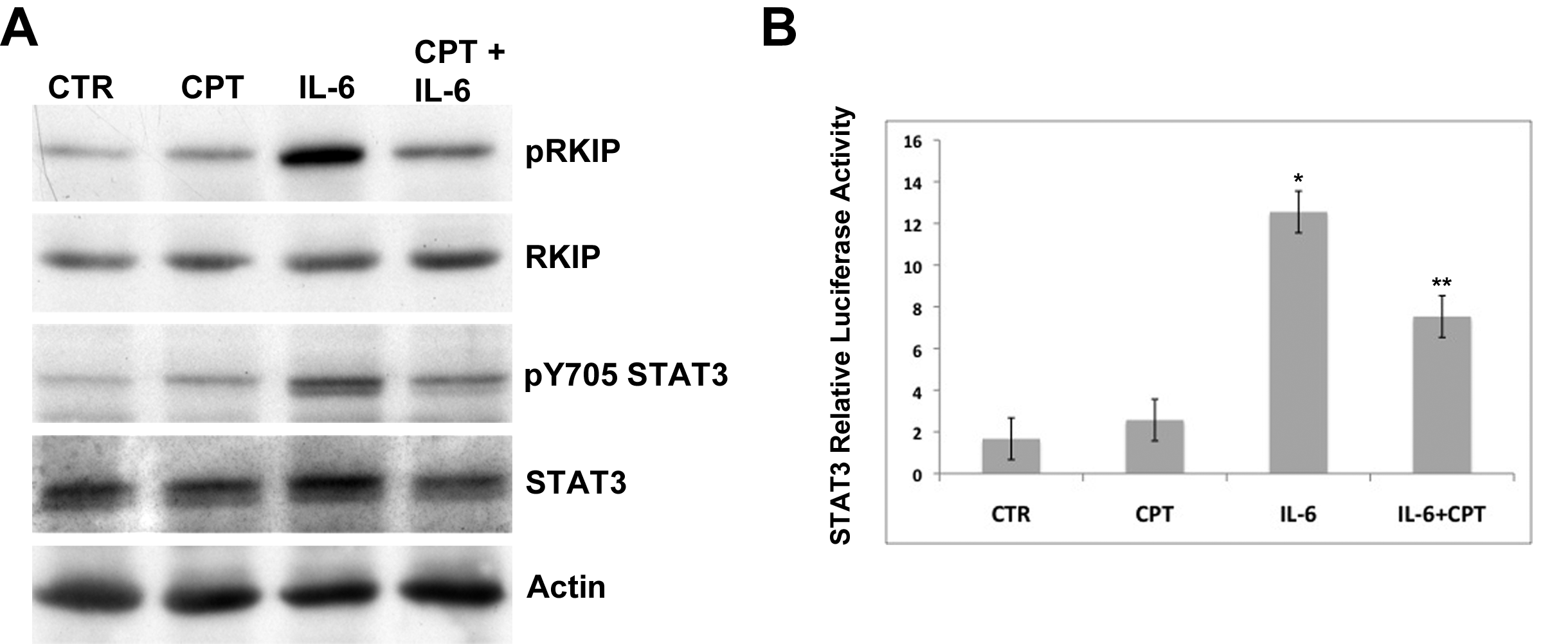

Supplement: Additional file 1: Figure S1 — Camptothecin blocks IL-6 mediated STAT3 activation in HT29 colon cancer cells. (A) Western blot analysis of the induction of RKIP and STAT3 phosphorylation after IL-6 treatment and subsequent reduction after treatment with 500 nM CPT; HT29 cells were treated with 500 nM CPT, 40 ng/ml IL-6 or the combination. (B) HT29 cells were transfected with an IRF-1 reporter plasmid to determine STAT3 activation. After 48 h, the cells were washed and treated with 40 ng/ml IL-6, 500 nM CPT, or the combination. After 24 h, samples were harvested and washed twice before being lysed and combined with a luciferase assay reporter. The data is reported as the mean +/− s.d. of 2 independent experiments performed in triplicate. A paired t-test was performed to analyze the increase in STAT3 transcription of IL- 6 treated experimental samples when compared to vehicle (CTR): *IL-6, p < 0.000017; or decrease when comparing IL-6 to samples treated with **IL-6 and CPT, p < 0.0008. [file 1471-2407-13-463-S1.tiff]
